# Pathogen discovery in AIDS-related lymphoma by high-throughput sequencing

**DOI:** 10.1186/1750-9378-7-S1-P45

**Published:** 2012-04-19

**Authors:** Akinyemi I Ojesina, Joonil Jung, Sharon Barouk, Ethel Cesarman, Matthew Meyerson

**Affiliations:** 1 Dana-Farber Cancer Institute, Broad Institute, Cambridge, MA, USA; 2 Weill Cornell Medical College, New York, NY, USA

## Background

Approximately 30% of AIDS-related lymphomas (ARL) are associated with infection by the EBV, and about 4% by the KSHV/HHV-8. It is likely that if other lymphomagenic pathogens exist, these associations would occur in the context of ARL. The advent of high throughput sequencing provides a unique opportunity to address this question. High throughput sequencing, followed by computational subtraction of human sequences was used for enrichment of candidate pathogenic sequences.

## Methods

Eleven primary tissues of ARL have been used to generate cDNA libraries. Out of these six were frozen specimens and five were formalin-fixed paraffin-embedded (FFPE). These libraries were subjected to high throughput Illumina sequencing to generate 30-60 million 76bp paired-end sequence reads per sample. Quality filtered reads were analyzed using our automated pipeline, *PathSeq*, which carries out several subtraction steps involving alignments to i) human genome sequence databases; ii) human transcriptome sequence databases; and iii) other vertebrate sequence databases. Residual sequence reads were then compared with microbial databases, either individually or as part of *de novo* assembled contigs.

## Results

Using both frozen and formalin-fixed paraffin-embedded (FFPE) tissues, we have identified unique sequences previously unassociated with hematological cancers and inflammatory diseases. In addition, our pathogen discovery pipeline works with both transcriptome and whole genome sequencing (WGS) data, and it is applicable to data across all high throughput sequencing platforms. Most notably, we are able to detect as low as 1 viral sequence per billion total sequences for WGS data, a sign of the sensitivity of our method. Among the known pathogens, we found 12423 sequences corresponding to EBV in the one case where the presence of this virus was also documented by EBER in situ hybridization. Three additional cases that were EBER-negative revealed EBV sequences, in the range of 3 to 351 reads, suggesting that the virus was present in tumor-infiltrating cells, rather than in the lymphoma. Eight cases had HIV sequences ranging from 1 to 403 reads, and one case had a single read corresponding to KSHV.

## Conclusions

We have developed an integrated pipeline, *PathSeq*, for pathogen discovery in both frozen and FFPE tissues using a high throughput sequencing-based computational subtraction process. The presence of >10,000 reads in the known EBV-positive case confirms the effectiveness of the method. Specific EBV and HIV sequences were seen emanating from tumor-infiltrating cells that will shed light on expression patterns of these viruses in this cellular compartment.

